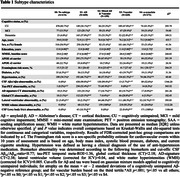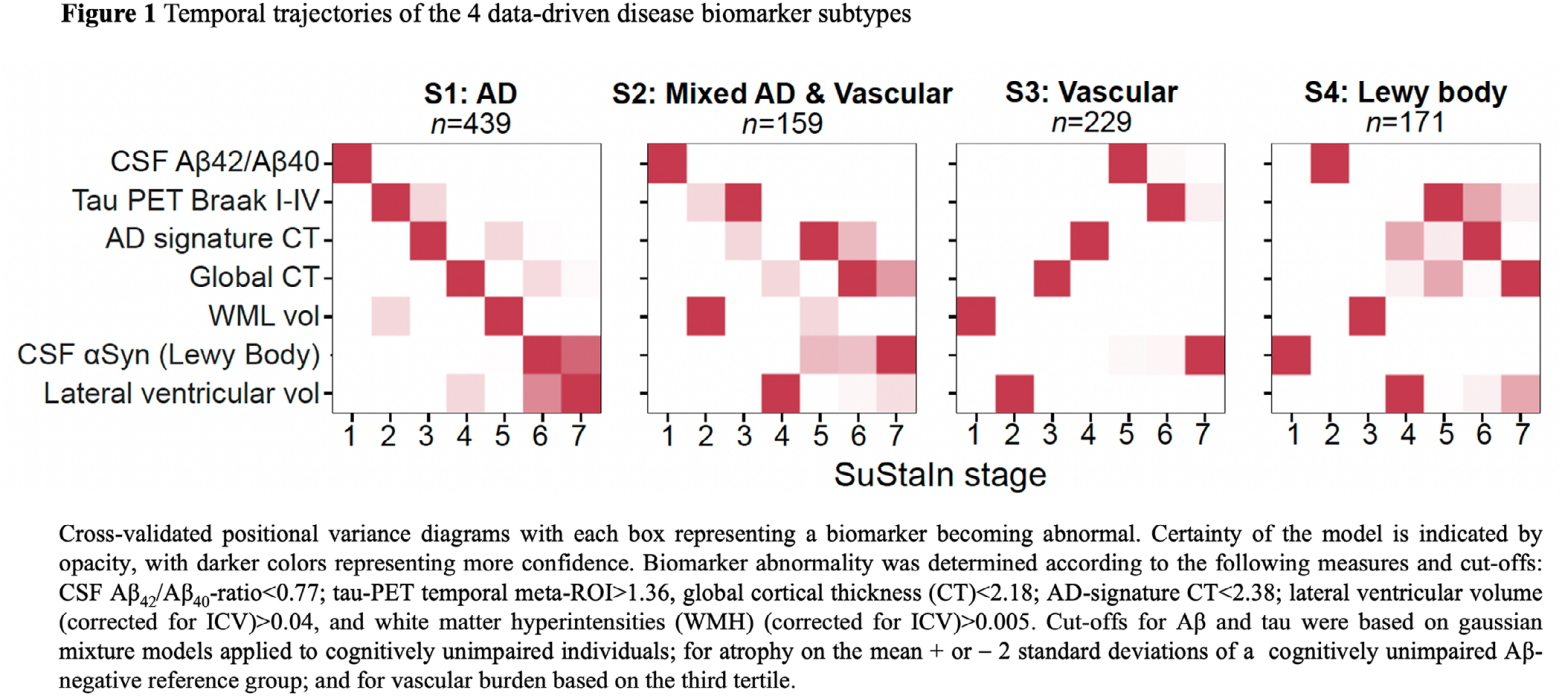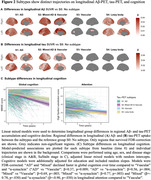# Data‐driven progression patterns of Aβ, tau, α‐synuclein and vascular pathology in a clinical population

**DOI:** 10.1002/alz.090303

**Published:** 2025-01-09

**Authors:** Sophie E. Mastenbroek, Lyduine E. Collij, Jacob W. Vogel, Alexandra L. Young, Olof Strandberg, Ruben Smith, Sebastian Palmqvist, Niklas Mattsson‐Carlgren, Frederik Barkhof, Rik Ossenkoppele, Oskar Hansson

**Affiliations:** ^1^ Amsterdam Neuroscience, Brain Imaging, Amsterdam Netherlands; ^2^ Department of Radiology and Nuclear Medicine, Vrije Universiteit Amsterdam, Amsterdam University Medical Center, location VUmc, Amsterdam Netherlands; ^3^ Clinical Memory Research Unit, Department of Clinical Sciences, Lund University, Lund Sweden; ^4^ Lund University, Lund Sweden; ^5^ Department of Clinical Sciences Malmö, SciLifeLab, Lund University, Lund Sweden; ^6^ Centre for Medical Image Computing, Department of Computer Science, University College London, London United Kingdom; ^7^ Clinical Memory Research Unit, Department of Clinical Sciences Malmö, Faculty of Medicine, Lund University, Lund Sweden; ^8^ Skåne University Hospital, Lund Sweden; ^9^ Memory Clinic, Skåne University Hospital, Malmö Sweden; ^10^ Wallenberg Center for Molecular Medicine, Lund University, Lund Sweden; ^11^ University College London, London United Kingdom; ^12^ Amsterdam Neuroscience, Neurodegeneration, Amsterdam Netherlands; ^13^ Alzheimer Center Amsterdam, Neurology, Vrije Universiteit Amsterdam, Amsterdam UMC location VUmc, Amsterdam Netherlands

## Abstract

**Background:**

With the development of disease modifying therapies targeting specific pathologies, accurate in vivo biomarkers have become increasingly important for disease classification. Recently, tests for neuronal α‐synuclein (Lewy body) pathology have become available, complementing pre‐existing tests for Alzheimer’s disease (AD) pathology (Aβ and tau fluid and PET biomarkers) and vascular disease (MRI). Here, we aimed to identify and characterize data‐driven pathology‐based subtypes using these biomarkers.

**Method:**

We included 1677 subjects from a prospective memory clinic cohort (BioFINDER‐2; age[mean±SD]=68.5±12.1; female=50.3%) ranging from cognitively unimpaired (CU) to dementia and including a variety of clinical diagnoses. We applied the data‐driven Event‐Based Subtype and Stage Inference (SuStaIn) model to dichotomized biomarkers of Aβ‐pathology (CSF‐Aβ), tau pathology (tau‐PET), neuronal α‐synuclein pathology (CSF‐α‐synuclein seeding aggregation assay) and vascular burden (MRI white matter lesions) to determine whether different biomarker sequences emerge within a clinical cohort based on the presence of different brain pathologies. Further, regional atrophy patterns (MRI) were included in the models.

**Results:**

Four disease‐trajectories best explained the data; 998 subjects with a confident assignment (mean probability=70%) were classified as “AD” (44.0%), “Mixed AD and Vascular” (15.9%), “Vascular” (22.9%), or “α‐synuclein” (17.2%) based on early‐stage pathology (Figure 1). 579 subjects were not classified and grouped as “No subtype” (mainly biomarker‐negative). The “Vascular” trajectory demonstrated early‐stage central and global atrophy, while “α‐synuclein” exhibited atrophy in late stages (Figure 1). Subjects in “Vascular” had higher levels of vascular risk factors and were more often APOE‐ε2 carriers. Subjects in “Vascular” and “α‐synuclein” were older and more frequently male, while those in “AD” and “Mixed” more frequently had a dementia diagnosis and APOE‐ε4 carriership (Table 1). Compared to unclassifiable subjects, “AD”, “Mixed”, and “α‐synuclein” showed faster widespread longitudinal Aβ accumulation and “AD” and “Mixed” demonstrated faster widespread longitudinal tau accumulation. “AD” and “Mixed” declined faster in global cognition over time compared to “Vascular” and “α‐synuclein” and “Mixed” and “α‐synuclein” in attention compared to “Vascular” (Figure 2).

**Conclusion:**

In a heterogenous clinical population, we identified four common pathology‐based disease trajectories resembling established clinico‐pathological relationships. Future analyses will compare disease‐patterns on longitudinal biomarkers. Disease classification might contribute to personalized medicine approaches in trial and clinical settings.